# Supplementary material for: Resilience to Cardiac Aging in Greenland Shark Somniosus microcephalus
Source: Aging Cell. 2026 Apr 23;25(5):e70505. doi: 10.1111/acel.70505 (PMC13105287; doi:10.1111/acel.70505)
Supplement: Supplementary file 1 — Figure S1: Representative of S. microcephalus ventricular wall. Black arrow: pericardial fibrotic capsule. Yellow arrow: compact myocardial layer. Green arrow: spongy myocardial layer. Yellow asterisks: coronary vessels. Blue scalebar: 500 μm. Figure S2: Representatives of S. microcephalus coronary cardiac fibrosis, green arrows: (A) female 341 cm; (B) female 341 cm; (C) male 310 cm. Figure S3: Representative examples of the (A) total tissue mask, (B) collagen mask, (C) overlay mask of Somniosus microcephalus (sample 7). Fibrosis percentage ratio is expressed as % (collagen/total area) as described in Materials and Methods section. Scalebar: 100 μm. Figure S4: Analysis of the 390 TL S. microcephalus specimen. Masson's trichrome staining of (A) compact and (B) spongy myocardial layer. Sudan black B of (C) compact and (D) spongy layer. Native lipofuscin of (E) compact and (F) spongy layer. IHC showing lysosomes and lipofuscin accumulation acquired with Airyscan: (G–J) compact and (K–N) spongy myocardial layer. Orange arrows: single examples of lysosomal Lamp1 and lipofuscin colocalization. Purple arrows: single examples of lipofuscin granules. Cyan: Hoechst. Green: Lamp1. White: lipofuscin. Black scalebar: 100 μm. White scalebar: 50 μm. [file ACEL-25-e70505-s004.pdf]

Supplementary figures

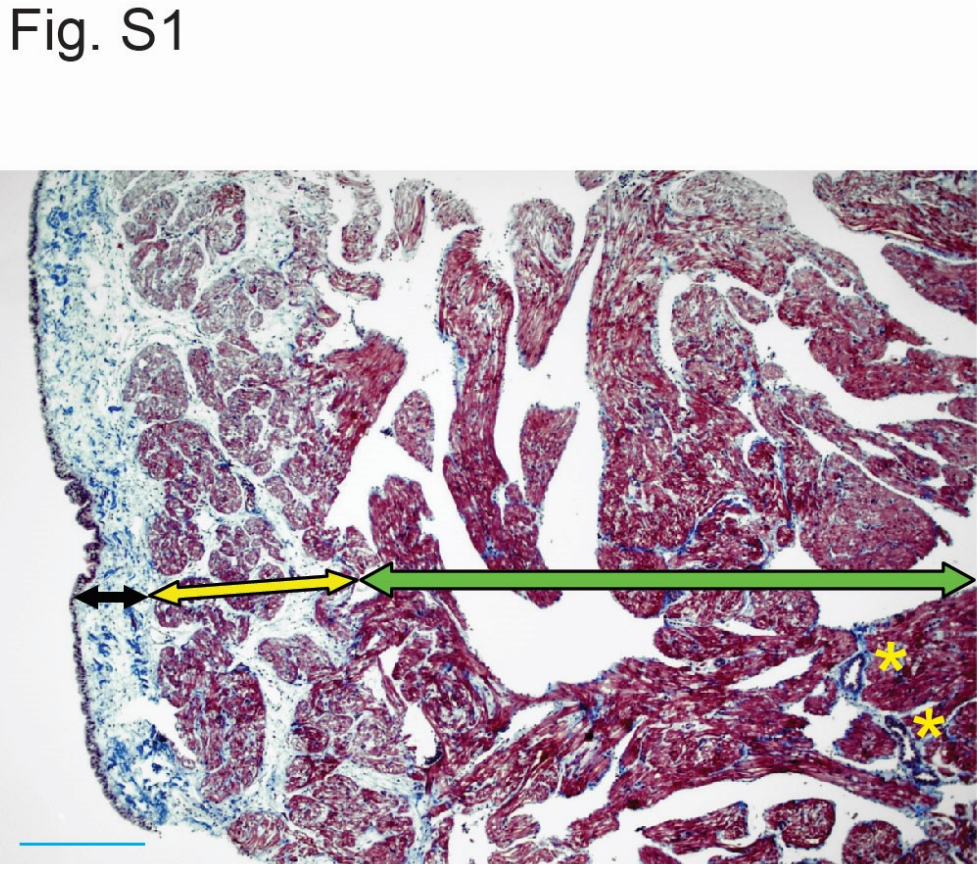

Figure S1: Representative of *S. microcephalus* ventricular wall. Black arrow: pericardial fibrotic capsule. Yellow arrow: compact myocardial layer. Green arrow: spongy myocardial layer. Yellow asterisks: coronary vessels. Blue scalebar: 500µm.

Fig. S2

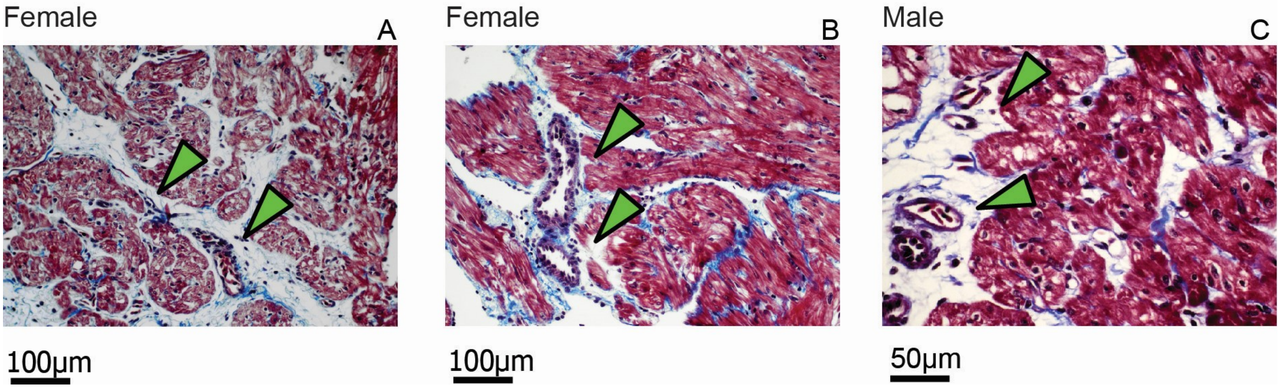

Figure S2: Representatives of *S. microcephalus* coronary cardiac fibrosis, green arrows: A) female 341cm; B) female 341cm; C) male 310cm.

Fig. S3

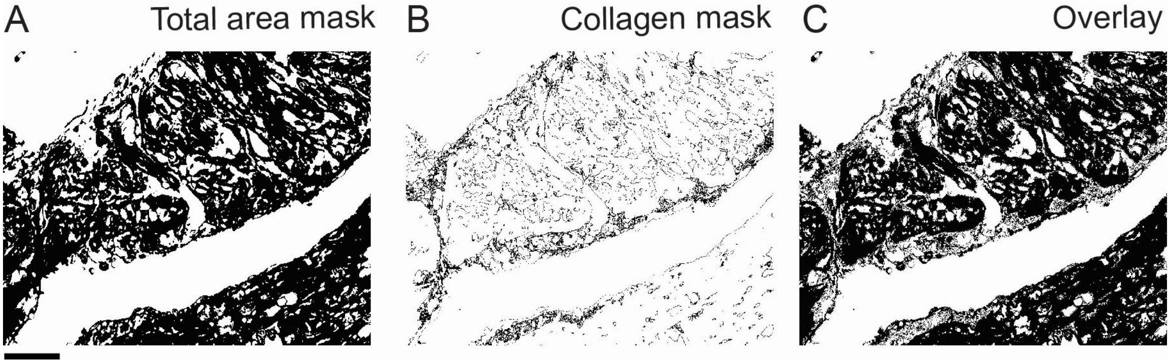

Figure S3: Representative examples of the A) total tissue mask , B) collagen mask, C) overlay mask of *Somniosus microcephalus* (sample 7). Fibrosis percentage ratio is expressed as % (collagen/total area) as described in Materials and Methods section. Scalebar: 100µm

Fig. S4

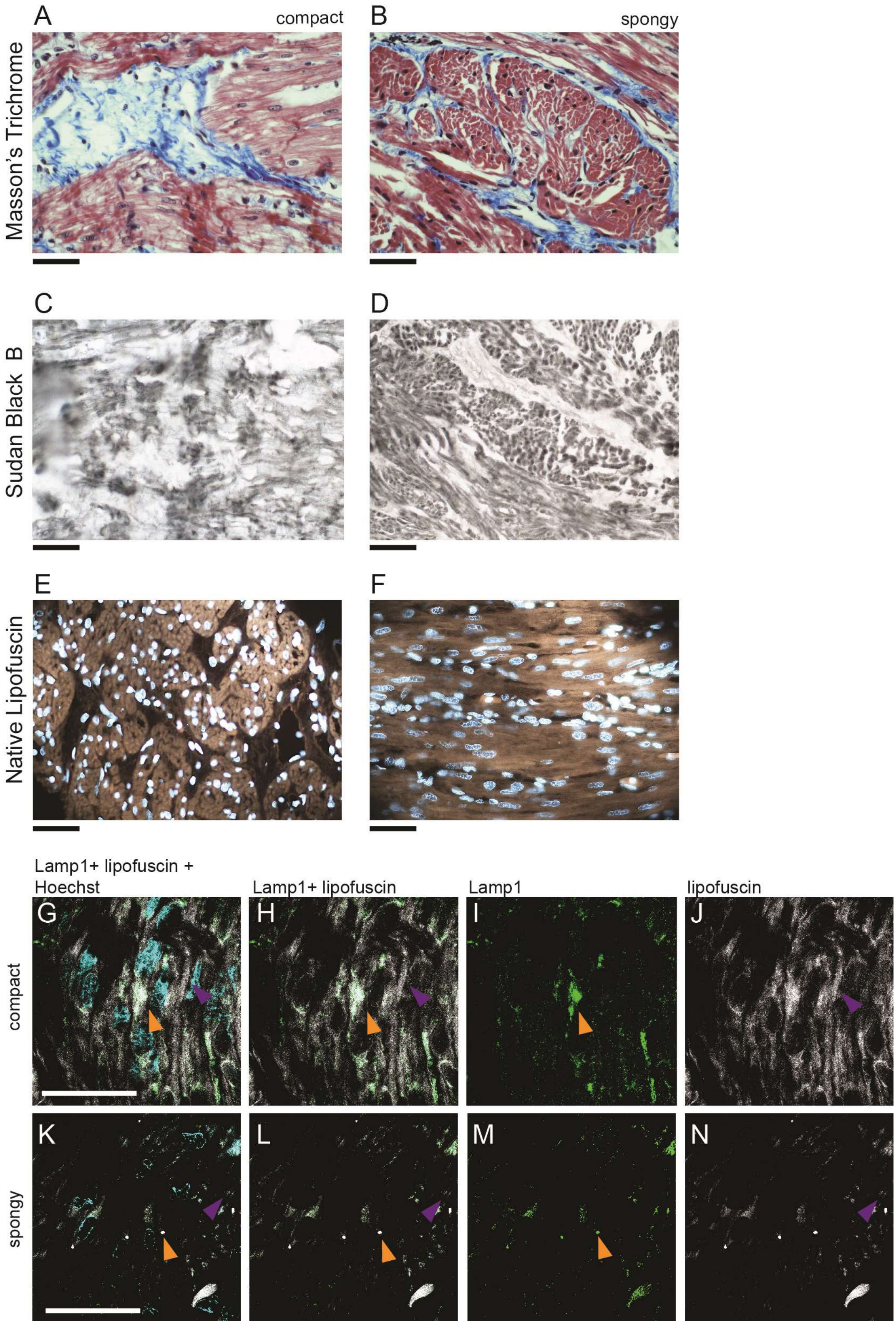

Figure S4: analysis of the 390 TL *S. microcephalus* specimen. Masson's trichrome staining of A) compact and B) spongy myocardial layer. Sudan black B of C) compact and D) spongy layer. Native lipofuscin of E) compact and F) spongy layer. IHC showing lysosomes and lipofuscin accumulation acquired with Airyscan: G–J) compact and K–N) spongy myocardial layer. Orange

arrows: single examples of lysosomal Lamp1 and lipofuscin colocalization. Purple arrows: single examples of lipofuscin granules. Cyan: Hoechst. Green: Lamp1. White: lipofuscin. Black scalebar: 100µm. White scalebar: 50µm.

Fig. S5

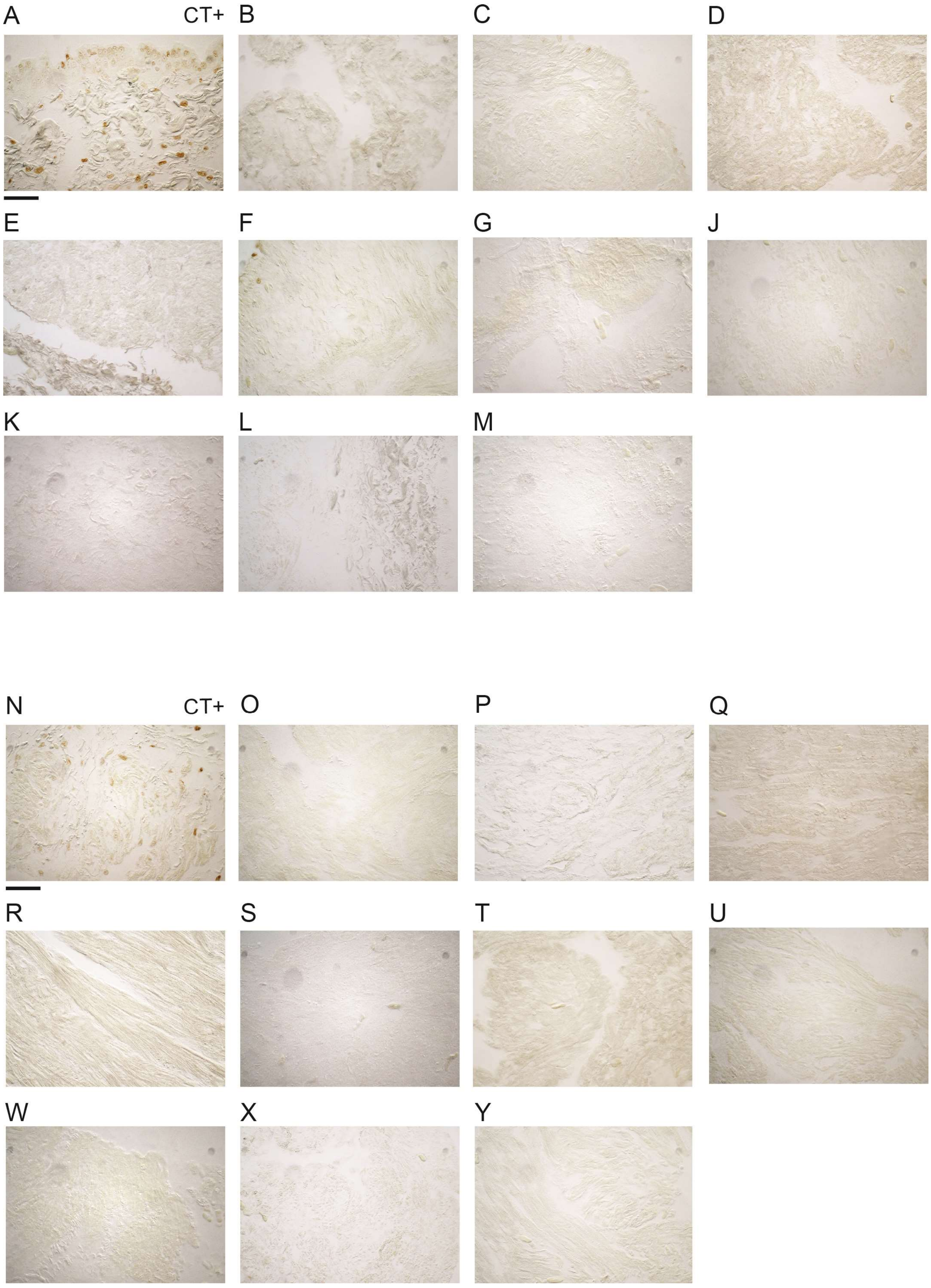

Figure S5: TUNEL assay of the *S. microcephalus* animals. A) positive control DNAse I treated compact myocardium of 341cm TL female. B–M) Compact myocardial layer of *S. microcephalus* animals, 1 to 10. N) Positive control DNAse I treated spongy myocardium of 341cm TL female. O–Y) Spongy myocardial layer of *S. microcephalus* animals, 1 to 10. Scalebar: 100μm.
