# Supplementary material for: Resilience to Cardiac Aging in Greenland Shark Somniosus microcephalus
Source: Aging Cell. 2026 Apr 23;25(5):e70505. doi: 10.1111/acel.70505 (PMC13105287; doi:10.1111/acel.70505)
Supplement: Supplementary file 2 — Table S1: acel70505‐sup‐0002‐TableS1.pdf. S. microcephalus parametres. F, female; M, male; IHC, immunohistochemistry Lamp1; EM, Electron Microscopy; N/A, Not Applicable. [file ACEL-25-e70505-s001.pdf]

Supplementary table 1

Samples	Sex	TL (cm)	Tissue processing	Masson's trichromic	Sudan Black B	Lipofuscin	IHC	EM
1	F	303	Paraffin	done	done	done	done	N/A
2	F	325	Paraffin	done	done	done	done	N/A
3	M	335	Paraffin	done	done	done	done	N/A
4	F	341	Paraffin	done	done	done	done	N/A
5	M	310	Paraffin	done	done	done	done	N/A
6	F	330	Paraffin	done	done	done	done	N/A
7	M	335	Paraffin	done	done	done	done	N/A
8	F	390	Paraffin	done	done	done	done	N/A
9	F	300	Paraffin	done	done	done	done	N/A
10	F	300	Paraffin	done	done	done	done	N/A
EM1	M	294	EM	N/A	N/A	N/A	N/A	done
EM2	F	310	EM	N/A	N/A	N/A	N/A	done
EM3	F	434	EM	N/A	N/A	N/A	N/A	done

Supplementary table 1: *S: microcephalus* parametres. F: female, M: male. IHC: immunohistochemistry Lamp1. EM: Electron Microscopy. N/A: Not Applicable.
