# Supplementary material for: Resilience to Cardiac Aging in Greenland Shark Somniosus microcephalus
Source: Aging Cell. 2026 Apr 23;25(5):e70505. doi: 10.1111/acel.70505 (PMC13105287; doi:10.1111/acel.70505)
Supplement: Supplementary file 3 — Table S2: acel70505‐sup‐0003‐TableS2.pdf. E. spinax parametres. F, female; M, male. [file ACEL-25-e70505-s003.pdf]

**Supplementary table 2**

| Samples | Sex | TL (mm) | Age ( years estimated) |
|---|---|---|---|
| 1 | F | 300 | 5.8 |
| 2 | F | 300 | 5.8 |
| 3 | F | 270 | 4.6 |
| 4 | F | 265 | 4.5 |
| 5 | F | 280 | 5.0 |
| 6 | F | 275 | 4.8 |
| 7 | M | 260 | 4.3 |

Supplementary table 2: *E: spinax* parametres. F: female, M: male.
